# Toward a standardized and interoperable imaging biomarker catalog

**DOI:** 10.1186/s13244-026-02304-6

**Published:** 2026-06-09

**Authors:** Pablo Rodríguez-Belenguer, Paula Doria-Borrell, Ángel Alberich-Bayarri, Raquel Perez-Lopez, Vicky Goh, Konstantin Nikolaou, Aad van der Lugt, Marion Smits, Luis Rodríguez-Rodríguez, Leonor Cerdá-Alberich, Luis Martí-Bonmatí

**Affiliations:** 1 Biomedical Imaging Research Group (GIBI230), Instituto de Investigación Sanitaria La Fe (IIS La Fe), Valencia, Spain; 2 Quibim—Quantitative Imaging Biomarkers in Medicine, Valencia, Spain; 3 Vall d’Hebron Institute of Oncology (VHIO), Barcelona, Spain; 4 Department of Cancer Imaging, School of Biomedical Engineering and Imaging Sciences, King’s College London, London, United Kingdom; 5 Department of Radiology, University of Tuebingen, Tuebingen, Germany; 6 Department of Radiology and Nuclear Medicine, Erasmus MC University Medical Center, Rotterdam, the Netherlands; 7 Brain Tumor Center, Erasmus MC Cancer Institute, Rotterdam, the Netherlands; 8 Musculoskeletal Pathology Group, Rheumatology Department, Hospital Clínico San Carlos, Instituto de Investigación Sanitaria del Hospital Clínico San Carlos (IdISSC), Madrid, Spain

**Keywords:** Imaging biomarker, FAIR, Catalog, Interoperable

## Abstract

**Introduction:**

Imaging biomarkers are quantifiable features extracted from medical images that indicate health status, disease characteristics, or treatment response. Their value depends on rigorous standardization and validation—efforts advanced by QIBA and EIBALL. Variability in existing inventories highlights the need for a FAIR (Findable, Accessible, Interoperable, Reusable)-compliant catalog to enable systematic discovery, comparison, and adoption.

**Materials and methods:**

We focused on defining the essential variables to describe imaging biomarkers across research, regulatory, and clinical settings. A three-phase approach was undertaken: (1) key resources—including FDA–NIH BEST, QIBA, EMA, ESR-EIBALL, relevant regulations, and scientific literature—were reviewed; (2) attributes were extracted and compared, with redundancies resolved by expert consensus; and (3) consolidated variables were organized into domains aligned with FAIR principles.

**Results:**

A unified biomarker descriptor set was established across five domains: core identification (imaging biomarker name, surrogation, clinical relevance), clinical context (main target, organ(s), disease/substrate, range(s), actionability), imaging and technical information (image modality, acquisition technique, technical parameters, extraction, association type, dimensionality, units), validation (robustness and use endorsed by publications, endorsed by professional societies, regulatory qualifications), and administrative data (repository, version/author). The catalog was tested across representative imaging biomarkers in inflammatory diseases, including ADC, FDG-PET, CT-based radiomics signatures, and Doppler ultrasound indices, demonstrating coherent descriptions across diseases and organs. Diagnostic and prognostic roles were clarified, transparency and reproducibility were promoted, and the need for context-adapted entries was shown.

**Conclusion:**

This work proposes a harmonized, scalable approach for cataloging imaging biomarkers. Consistent descriptors across contexts facilitate integration into research, regulatory, and clinical workflows.

**Critical relevance:**

This work proposes a unified catalog structure for imaging biomarkers that improves standardization, comparability, and reusability across clinical, research, and regulatory domains.

**Key Points:**

- Lack of standardization limits the integration of imaging biomarkers into research, regulatory, and clinical workflows.
- A harmonized catalog was developed using unified descriptors across the main identified domains.
- This structure enhances traceability, cross-disease comparability, and regulatory readiness of imaging biomarkers.

**Graphical Abstract:**

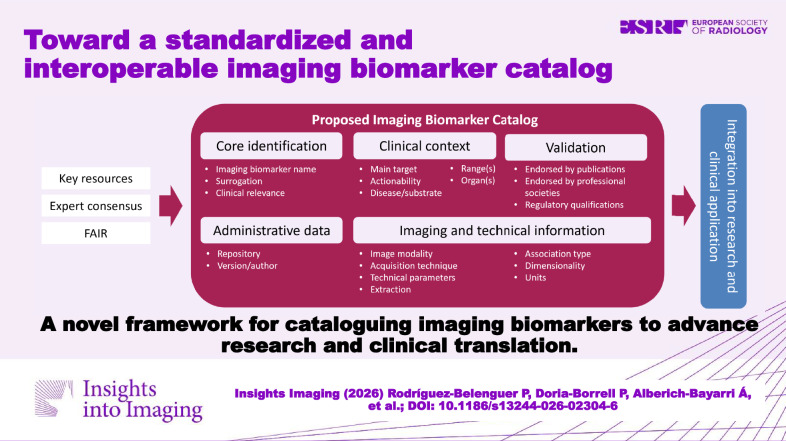

## Introduction

Biomarkers are a cornerstone of clinical research and translational precision medicine, providing objective and quantifiable surrogates of biological states or of future clinical events. They play critical roles in diagnosis, treatment response prediction, prognosis, and disease monitoring, and their increasing use in clinical decision-making has amplified the need for clear classification and standardization across disciplines. A widely cited description established by the Biomarkers Definitions Working Group, convened by the U.S. National Institutes of Health (NIH), defines a biomarker as “a characteristic that is objectively measured and evaluated as an indicator of normal biological processes, pathogenic processes, or pharmacologic responses to a therapeutic intervention” [1]. While objectivity is essential, biomarkers often act as measurable proxies for biological processes or outcomes.

Addressing these limitations, the U.S. Food and Drug Administration (FDA) and the NIH jointly developed the “Biomarkers, Endpoints, and other Tools” (BEST) resource, a harmonized framework for biomarker terminology and classification that emphasizes the importance of defining their context of use [2].

Within this context, imaging biomarkers represent a growing and dynamic subset of the biomarker landscape. They enable non-invasive assessment of organs, tissues, or lesions and can be repeatedly measured over time, providing insights into disease progression or therapy response while supporting multiple functional roles across the biomarker spectrum. For effective application in clinical practice and trials, imaging biomarkers must be understandable, accessible, precise, accurate, and reproducible [3]. Ensuring these criteria are consistently met remains challenging, as their utility depends not only on what they measure but also on the accessibility and reliability of the measurement process. Addressing this requires robust frameworks for validation, standardization, and interpretation, particularly in a field rapidly evolving through advances in imaging technologies, quantitative analysis, and computational methods [4].

In response to these demands, several professional societies and research alliances have launched coordinated efforts to promote best practices and improve the reliability and clinical adoption of imaging biomarkers. Key international initiatives such as Quantitative Imaging Biomarkers Alliance (QIBA), European Imaging Biomarkers Alliance (EIBALL), and American College of Radiology Imaging Network (ACRIN) have contributed to harmonizing terminology and standardizing acquisition and analysis protocols across imaging modalities [5–8]. However, many essential elements needed to characterize imaging biomarkers—such as organ-specific descriptors, surrogates, validation criteria, regulatory status, and study provenance—remain scattered across individual publications. As a consequence, there is a need to harmonize and centralize key information on imaging biomarkers: descriptors should be standardized across organ systems, surrogates should be standardized, validation criteria need to be harmonized and methodologically justified, regulatory endorsement ought to be systematically reported, and essential elements such as version control, authorship attribution, and update traceability must be documented to ensure transparency and long-term usability. To address these challenges, a dedicated catalog is needed to systematically reunite key information on imaging biomarkers in an accessible and standardized format, thereby facilitating comparison, interpretation, and practical application. In this context, the Findable, Accessible, Interoperable, and Reusable (FAIR) Guiding Principles provide a valuable framework for ensuring that data can be effectively shared and reused. Here, interoperability is understood as both syntactic, enabled by a shared and structured set of descriptors, and semantic, ensured through consistent definition and interpretation of those descriptors across contexts. Accordingly, applying FAIR principles to imaging biomarker cataloging supports consistent discovery, integration, and reuse of biomarkers across research, regulatory, and clinical contexts [9].

Building on these principles, we aim to define the essential requirements of an interoperable and comprehensive imaging biomarkers catalog. The approach was designed to facilitate comparison between resources, evaluate their quality, and overcome current limitations in accessibility, standardization, and integration. Ultimately, the catalog seeks to support more effective use of imaging biomarkers in research, regulatory assessment, and clinical decision-making.

## Methods

### Development of the imaging biomarker catalog

To select the catalog core components, a rationale-driven structure approach was defined. The aim was not to compile a list of biomarkers, but rather to establish the essential variables needed to describe them in a consistent and meaningful way across research, regulatory, and clinical contexts.

**Fig. 1:**
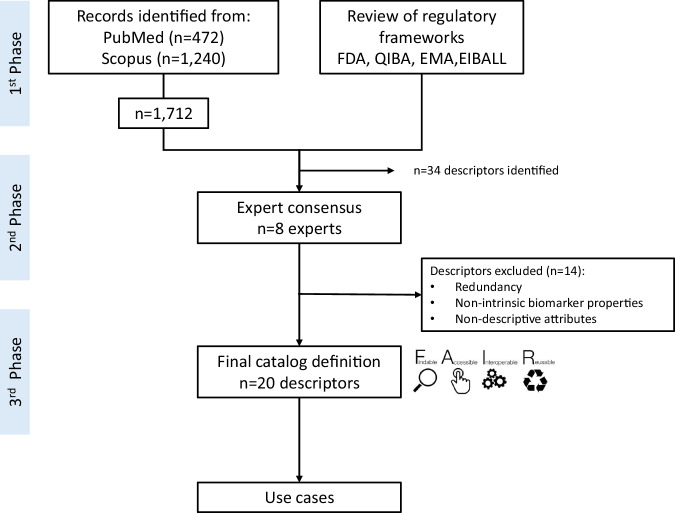
Overview of the three-phase methodology used to define the imaging biomarker descriptor catalog. Phase 1 comprised a structured review of the literature (PubMed and Scopus) and regulatory frameworks (FDA, QIBA, EMA, EIBALL). Phase 2 involved expert consensus to refine and select relevant descriptors. Phase 3 resulted in a final catalog of descriptors structured for use case application (ADC), with exclusions based on redundancy, non-intrinsic biomarker properties, and non-descriptive attributes

A PRISMA-like flow diagram summarizing the identification, screening, and inclusion of sources is provided in Fig. 1. The three phases were designed with distinct objectives: Phase 1 involved selecting reference sources and extracting descriptors; Phase 2 finalized the descriptor list through expert consensus; Phase 3 organized the resulting variables into a coherent, interoperable structure.

### Phase 1: Literature review and source extraction

Literature searches were performed in PubMed and Scopus, covering publications from January 2015 to July 2025. The search strategy combined controlled vocabulary and free-text terms related to imaging biomarkers and classification frameworks, including:

*“imaging biomarker” AND (descriptor* OR classification OR framework OR catalog OR inventory) *“quantitative imaging biomarker” AND (definition OR standardization)*

In addition, authoritative reference frameworks and regulatory documents were identified through targeted searches of organizational websites and prior knowledge, including BEST Resource developed by the FDA–NIH Biomarker Working Group [2], the QIBA Profiles published by the Radiological Society of North America (RSNA) [10], the Imaging Biomarkers Inventory from the EIBALL [6], and the regulatory documentations from the FDA Biomarker Qualification Program [11] and the European Medicines Agency (EMA) [12]. Descriptors were extracted from the reviewed sources.

### Phase 2: expert consensus

The selection and consolidation of core catalog variables was performed by a multidisciplinary working group, comprising eight experts from radiology, imaging biomarker development, clinical research, and regulatory sciences. The group contributed by reviewing sources, resolving semantic overlaps, and reaching consensus on the inclusion and definition of variables.

Descriptors were excluded when they:

1. Were redundant with existing variables,
2. Reflected application-dependent usage (e.g., monitoring, pharmacodynamic response) rather than intrinsic biomarker properties, or
3. Represented performance, validation, or implementation of characteristics (e.g., bias, reproducibility, robustness) rather than descriptive attributes.

Consensus was achieved through discussion and iterative refinement rather than formal voting procedures. All authors reviewed and approved the final set of variables.

### Phase 3: final catalog definition

In the final phase, the consolidated variables were organized into a structured set of fields proposed for the catalog. These were grouped into thematic domains (such as technical parameters, clinical interpretation, validation, and endorsement), refined for clarity and coherence, and aligned with the FAIR principles to ensure future interoperability, traceability, and reuse.

Terminology for quantitative imaging biomarkers followed the nomenclature proposed by the QIBA, with mathematical formulations derived from the corresponding methodological literature. Radiomic feature terminology followed the standardized definitions of the image biomarker standardization initiative (IBSI), and the corresponding equations were obtained from the PyRadiomics open-source library [13], which implements feature definitions consistent with IBSI standards.

The catalog structure has been implemented in a dedicated web-based platform and will be made publicly accessible upon finalization [14].

### Use cases of imaging biomarkers

We present use cases involving four imaging biomarkers representative of different modalities and analytical approaches—apparent diffusion coefficient (ADC) in bowel inflammation, Fluorodeoxyglucose - Positron Emission Tomography (FDG-PET) standardized uptake value (SUV) in large vessel vasculitis, Computed Tomography (CT)-based radiomics signatures in systemic sclerosis–associated interstitial lung disease, and Doppler ultrasound (US) vascularity index in rheumatoid arthritis—all developed within the Redes de Investigación Cooperativa Orientadas a Resultados en Salud (RICORS) network (https://www.isciii.es/financiacion/ricors/ricors-rei) [15].

Reference sources were selected a priori based on predefined criteria aimed at ensuring relevance across research, regulatory, and clinical contexts. Scientific literature was reviewed, selecting a high level of evidence according to Martí-Bonmatí et al [16]. These criteria were used to prioritize sources and publications that demonstrated methodological rigor, external validation, and relevance to clinical or regulatory decision-making, while studies with limited validation or purely exploratory scope were excluded. To be classified as a high level of evidence, the literature had to meet one or more of the following criteria:

(i) Data derived from meta-analyses, systematic reviews, or from (multiple) randomized trials with high quality.
(ii) Large retrospective observational studies or in silico clinical trials with external validation.
(iii) Well-defined reference standards and controlled biases.
(iv) The described technique improves healthcare pathways (tests, treatment, and hospitalization) or decreases costs per patient.
(v) The level is graded down to Moderate if there are limiting biases or inconsistencies between studies.

All information compiled from the selected biomarker sources was subsequently incorporated into the proposed imaging biomarker catalog by populating the corresponding descriptors.

## Results

### Proposed catalog structure

The structure of the imaging biomarker catalog was defined through a selection process aimed at identifying descriptors that are intrinsic, stable, and broadly applicable across clinical and research contexts. Rather than capturing all analytical or performance-related properties, the catalog focuses on descriptive variables that support interpretability, interoperability, and reuse.

Several descriptors commonly reported in the imaging biomarker literature were excluded. As summarized in Supplementary Table 1, variables were omitted when they were redundant, application-dependent, or related to performance, validation, or implementation characteristics (e.g., reproducibility, robustness, bias, diagnostic accuracy metrics). Such properties are highly dependent on acquisition protocols, analysis pipelines, and clinical context, and therefore do not constitute stable attributes suitable for a general-purpose catalog.

Functional classifications such as monitoring, pharmacodynamic response, or susceptibility/risk were also excluded as standalone descriptors, as they describe how a biomarker is used in specific scenarios rather than its inherent characteristics. To preserve alignment with established frameworks while avoiding redundancy, functional roles were consolidated into four primary categories—diagnostic, prognostic, predictive, and response—captured through the Main target descriptor.

Following this process, a structured set of descriptors was defined to represent imaging biomarkers in a consistent, interpretable, and reusable format. Table 1 presents the variables that constitute the structure. For each descriptor, data type, and cardinality were defined to support unambiguous interpretation and machine-actionable use. Each row corresponds to a descriptor capturing relevant information about a biomarker, including its clinical role, technical derivation, validation status, regulatory recognition, and administrative traceability.

**Table 1:** Proposed descriptors of an imaging biomarker catalog [16, 19, 24, 25]




The color palette indicates the five domains for classifying the biomarker descriptors: core identification (pink), clinical context (blue), imaging and technical information (green), validation (orange), and administrative data (yellow)

^*^Cardinality is expressed as minimum–maximum occurrence: 1 = exactly one value required; 0..1 = optional, at most one value; 1..n = one or more values required; 0..n = optional, multiple values allowed

The rationale for the descriptors in Table 1 is detailed in Supplementary Table 2. In summary, descriptors related to biomarker identification and clinical context ensure clinical interpretability and linkage to patient-centered outcomes. Imaging and technical descriptors provide transparency regarding biomarker derivation and support methodological consistency and reproducibility across studies and platforms. Quantitative descriptors (e.g., units and ranges) enable unambiguous measurement and facilitate comparison across cohorts, while actionability links biomarker values to potential clinical decisions. Descriptors related to evidence, governance, and implementation—such as publications, professional society endorsement, regulatory qualifications, repository linkage, and version control—support credibility, traceability, regulatory alignment, and long-term maintenance of the catalog.

Collectively, these descriptors provide the minimum information required by clinicians, researchers, and regulators, reflecting the multidisciplinary consensus process described in the Methods section. To preserve compatibility with established frameworks while avoiding duplication, the functional biomarker types defined in the BEST framework were consolidated into four categories—diagnostic, prognostic, predictive, and response—captured through the Main target descriptor. Monitoring biomarkers are included within the response category, safety biomarkers within the predictive category, and susceptibility or risk biomarkers within the prognostic category.

**Fig. 2:**
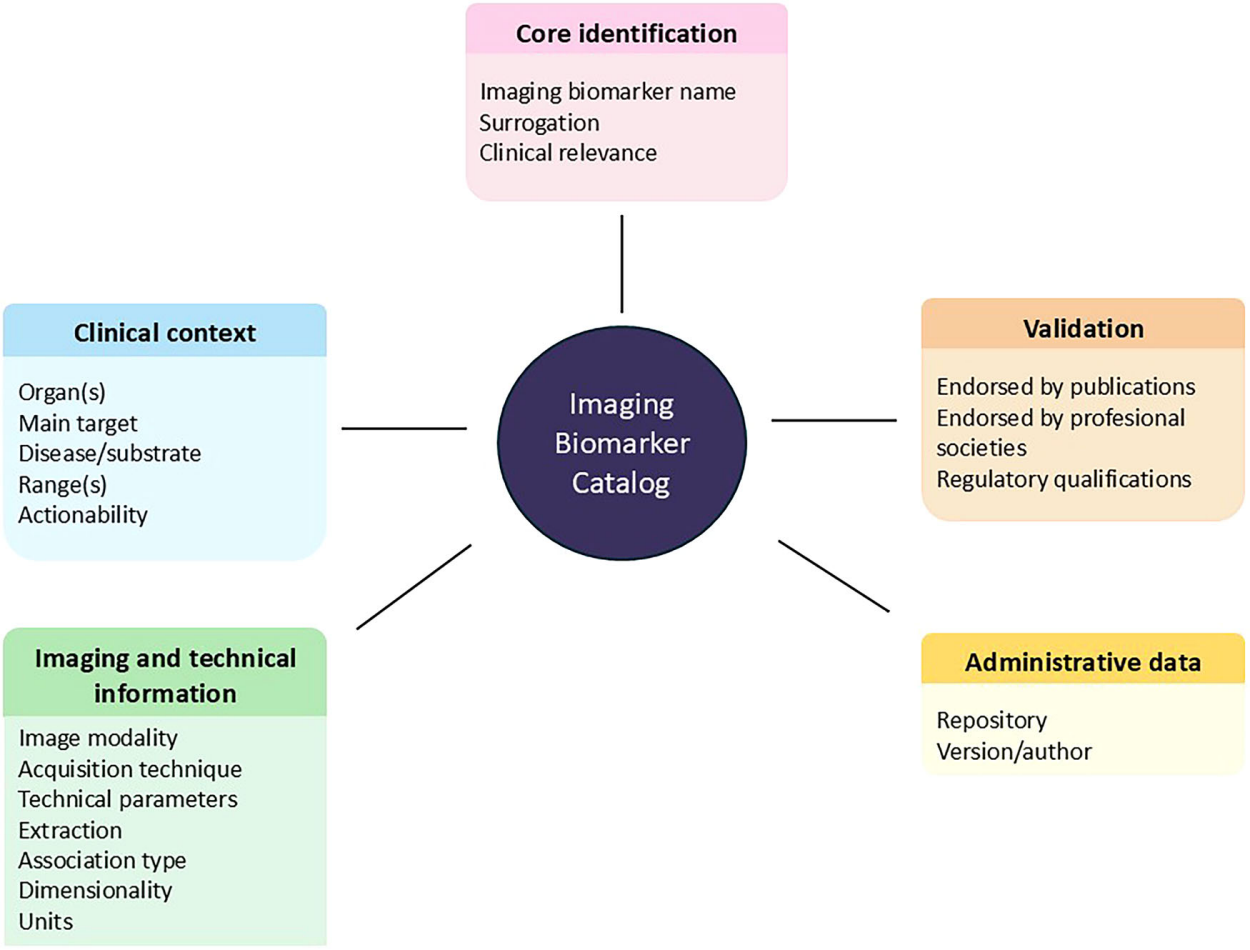
Conceptual organization of the imaging biomarker catalog

As summarized in Fig. 2, the descriptors are organized into five domains: core identification, clinical context, imaging and technical information, validation, and administrative data. Although conceptually grouped, all variables are implemented within a unified tabular structure to support consistent implementation, interoperability, and reuse.

### Use cases across inflammatory diseases

To illustrate the practical application and scalability of the proposed catalog structure, four representative imaging biomarkers were selected across different inflammatory disease contexts: the ADC in bowel inflammation; FDG-PET SUV metrics in large vessel vasculitis; CT-based radiomics signatures in systemic sclerosis–associated interstitial lung disease; and Doppler US vascularity index in rheumatoid arthritis. These biomarkers span magnetic resonance, nuclear medicine, CT, and US modalities, and represent both deterministic quantitative measurements and composite, model-derived metrics.

ADC is a quantitative magnetic resonance (MR) biomarker reflecting microstructural tissue changes associated with inflammation, edema, or altered cellularity [10]. Derived from diffusion-weighted MR images, ADC quantifies water mobility within tissues and acts as a surrogate for microarchitectural processes that are not directly measurable in vivo. FDG-PET SUV metrics quantify tissue glucose metabolic activity and are widely used to assess inflammatory burden in vascular territories. In this example, CT-based radiomics signatures capture textural heterogeneity associated with inflammatory and fibrotic remodeling, while Doppler US vascularity index quantifies synovial microvascular flow as a surrogate of inflammatory hyperemia.

Table 2 presents the descriptors that remain intrinsic and stable for each biomarker, independent of specific disease context. These include the biological surrogate, image modality, acquisition technique, technical parameters, extraction methodology, association type, dimensionality, units, and repository availability.

**Table 2:**
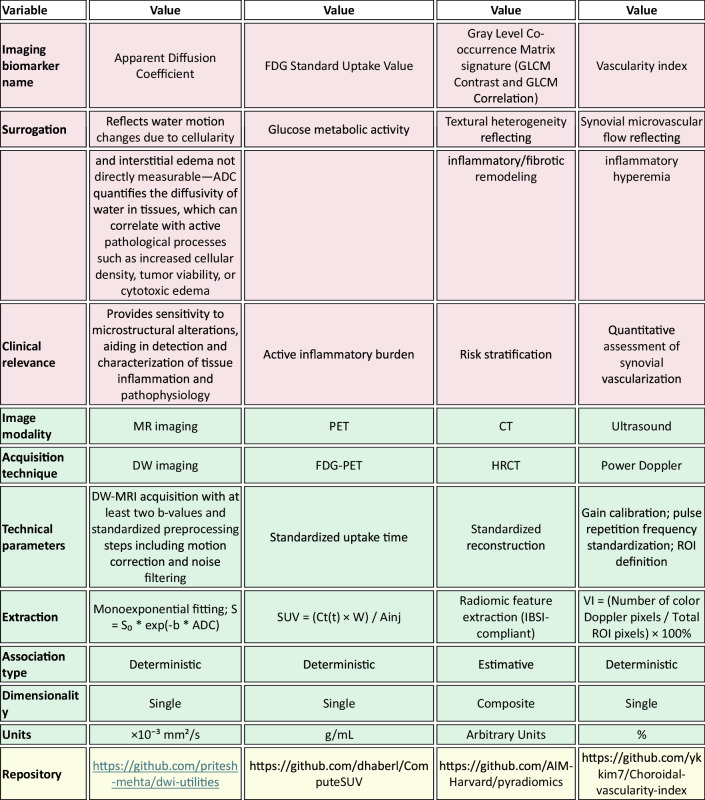
Intrinsic descriptors of representative imaging biomarkers across modalities

The technical backbone of each biomarker reflects modality-specific acquisition and computational processing steps. For example, ADC estimation requires diffusion-weighted MR acquisitions with at least two b-values and monoexponential fitting. SUV calculation is based on tissue activity concentration normalized to injected dose and body weight. Radiomics extraction follows standardized feature definitions and modeling approaches, and the Doppler vascularity index is derived from the proportion of color pixels within a region of interest. These descriptors ensure methodological transparency and reproducibility across institutions.

Table 3 displays the context-specific descriptors for each biomarker within its inflammatory disease application. For example, ADC values are typically reduced in inflamed bowel segments in Crohn’s disease. FDG-PET in large vessel vasculitis relies on vessel-to-liver SUV ratios and visual grading relative to hepatic uptake to identify active inflammation [17, 18]. Radiomics models use composite scores with cut-offs derived from training and validation cohorts, while Doppler US vascularity indices quantify synovial vascularization to assess inflammatory activity.

**Table 3:**
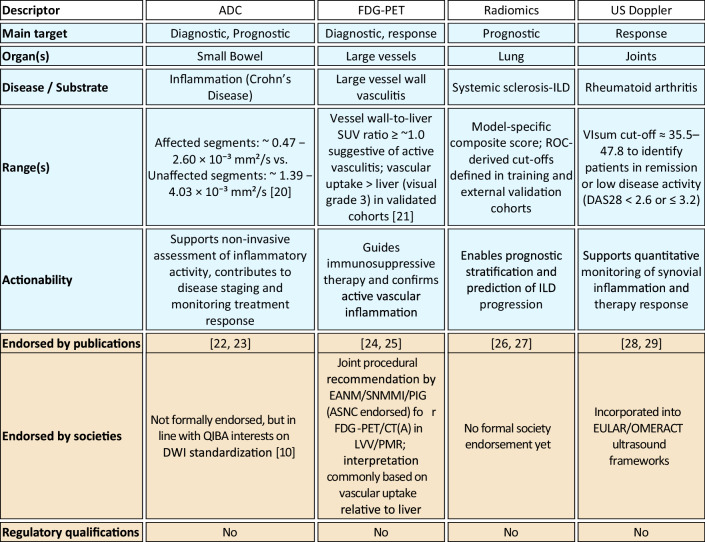
Shared intrinsic descriptors of representative imaging biomarkers. [26–33]

Evidence supporting these applications is reflected in the cited publications, including studies with external validation and defined reference standards. Selected biomarkers align with professional society recommendations, including QIBA guidance for diffusion-weighted magnetic resonance imaging (MRI) standardization and joint procedural recommendations for FDG-PET in large vessel vasculitis. However, no formal regulatory qualifications currently exist for these biomarkers in the inflammatory contexts presented.

## Discussion

The development of a structured, interoperable, and standardized catalog of imaging biomarkers addresses a critical gap in current biomedical. Major challenges persist in the absence of standard descriptors, harmonized validation criteria, systematic reporting of regulatory status, and transparent mechanisms for version control, authorship attribution, and update traceability. To overcome these limitations, we propose a cataloging framework that integrates technical, clinical, and regulatory descriptors into a coherent and reusable structure. The approach is explicitly aligned with the FAIR Guiding Principles, ensuring that imaging biomarker data are FAIR, and thereby supporting their effective application in research, regulatory contexts, and clinical practice.

Several frameworks and initiatives have previously addressed different aspects of biomarker development and classification. The BEST framework provides a widely adopted cross-domain functional classification of biomarkers. It distinguishes diagnostic, prognostic, predictive, monitoring, pharmacodynamic-response, safety, and susceptibility/risk biomarkers. While this classification offers a valuable conceptual foundation, these functional roles are not mutually exclusive and often depend on clinical context, disease stage, and intended use.

Other initiatives have focused primarily on technical standardization. In this sense, the mission of the QIBA was to “improve the value and practicality of imaging biomarkers by reducing variability across devices, patients, and time” [19]. QIBA achieved this goal through the development of modality-specific Profiles that define acquisition protocols and quantitative performance claims across imaging modalities such as MRI, CT, PET, and US. While these technical profiles are essential to facilitate reproducible and reliable quantitative imaging measurements, they do not provide a structured system for classifying imaging biomarkers themselves, nor do they systematically integrate clinical context, regulatory status, or governance-related information.

Building on these efforts, the EIBALL was established to support the development, standardization, and clinical integration of imaging biomarkers, following a brainstorming meeting of the QIBA European Task Force and positioning EIBALL as a European extension of QIBA’s activities [20]. A flagship outcome of EIBALL is the Imaging Biomarkers Inventory, an online resource that catalogs imaging biomarkers by disease and modality [6]. Using imaging biomarkers such as ADC, FDG-PET SUV, CT-based radiomics signatures, and Doppler US vascularity index as illustrative examples, the inventory demonstrates both the value and the limitations inherent to such an approach. Because the inventory is organized primarily by organ system, descriptors and the level of detail provided vary across sections (e.g., breast, prostate, pancreas), which can complicate systematic comparison. Moreover, elements that are highly relevant for clinical translation—such as acquisition protocols, extraction methodology, and quantitative ranges—are not uniformly included. Updates to individual sections, such as the prostate biomarker revision in April 2025, highlight the dynamic nature of the inventory but also underscore the importance of transparent versioning, authorship attribution, and traceability of modifications to ensure long-term reliability.

Taken together, these initiatives illustrate that while key information required to characterize imaging biomarkers exists, it remains fragmented across publications, inventories, and technical standards, and is often reported with variable depth and structure. This fragmentation limits systematic comparison, reuse, and integration across clinical and research contexts, and highlights the need for a harmonized, domain-independent framework capable of centralizing descriptive, technical, and governance-related information in a consistent manner.

In contrast to BEST, which provides a general functional classification applicable across biomarker types, the proposed catalog focuses specifically on imaging biomarkers and emphasizes intrinsic and stable descriptive attributes while allowing multiple functional roles to be captured through a single Main target descriptor. Unlike QIBA, which concentrates on technical performance and standardization, the proposed framework integrates clinical context, validation status, regulatory recognition, and administrative governance within a unified structure. Compared with the EIBALL Imaging Biomarkers Inventory, the proposed catalog introduces a harmonized, domain-independent set of descriptors designed to support consistency, comparability, and reuse across organ systems and clinical contexts.

Importantly, this also implies that the application of imaging biomarkers in clinical practice may depend on factors that are not fully captured by standardized descriptors alone. In particular, the extrapolation of quantitative imaging biomarkers across institutions and clinical scenarios may be influenced by image quality and by how the biomarker is assessed and interpreted in routine settings. Variability in acquisition protocols may affect the technical validity of the measurement, while differences in reader experience and familiarity with the biomarker may influence how these measurements are derived and applied clinically. For example, in prostate MRI, standardized image quality assessment frameworks such as the PI-QUAL score have been proposed to help ensure that quantitative parameters are derived from technically adequate studies [21]. Similar challenges have been reported in breast and bladder imaging, where variability in image acquisition and interpretation may influence biomarker reliability and clinical applicability [22, 23].

In a nutshell, the proposed structured catalog provides not only a standardized and harmonized set of descriptors, but also a model that supports comparability, traceability, and reuse of imaging biomarker information. By enabling multiple clinical contexts to be captured for a single biomarker, this approach offers a model designed to enhance transparency, scalability, and semantic robustness. Rather than replacing existing initiatives, it is intended to complement and strengthen them through a framework that is both clinically meaningful and technically interoperable.

Despite these strengths, this work has limitations to be acknowledged. First, the proposed catalog represents a conceptual and structural framework that has not yet been formally validated for completeness, inter-annotator consistency, or long-term adoption across institutions. While the descriptors were defined through multidisciplinary expert consensus, systematic evaluation involving external stakeholders—such as broader clinical communities, industry partners, or regulatory bodies—has not yet been conducted.

In addition, usability studies assessing annotation burden, clarity of definitions, and ease of implementation in real-world settings were beyond the scope of this study. Likewise, large-scale integration pilots with existing clinical data infrastructures, registries, or imaging repositories have not yet been performed. These evaluations will be essential to assess practical feasibility, semantic consistency across users, and the capacity of the catalog to support automated or semi-automated workflows.

Future work will therefore focus on external validation of the catalog structure, refinement of descriptors based on user feedback, and pilot implementations within clinical and research environments. These efforts will be critical to ensure that the proposed framework evolves from a structured proposal into a widely adopted and sustainable resource for imaging biomarker harmonization.

## Conclusion

A structured and interoperable imaging biomarkers catalog was developed, building on modern standards of data. This descriptor-harmonized catalog enables systematic comparison across anatomical sites, lesions, or range, among other variables, ensuring that biomarkers are described consistently. The proposal includes mechanisms to ensure traceability and enable comparisons, laying out the foundation for a clinically meaningful and technically robust resource that supports research, regulatory assessment, and clinical application.

## ELECTRONIC SUPPLEMENTARY MATERIAL

Supplementary information

## Abbreviations

ADC: Apparent Diffusion Coefficient
BEST: Biomarkers, endpoints, and other tools
CT: Computed tomography
EIBALL: European Imaging Biomarkers Alliance
EMA: European Medicines Agency
ESR: European Society of Radiology
FAIR: Findable, accessible, interoperable, and reusable
FDA: U.S. Food and Drug Administration
FDG-PET: Fluorodeoxyglucose-positron emission tomography
IBSI: Image biomarker standardization initiative
MR: Magnetic resonance
MRI: Magnetic resonance imaging
NIH: National Institutes of Health
PET: Positron emission tomography
QIBA: Quantitative imaging biomarkers alliance
RICORS: Redes de Investigación Cooperativa Orientadas a Resultados en Salud
RSNA: Radiological Society of North America
SUV: Standardized uptake value
US: Ultrasound

## References

[1] Biomarkers Definitions Working Group (2001) Biomarkers and surrogate endpoints: preferred definitions and conceptual framework. Clin Pharmacol Ther 69:89–95. 10.1067/mcp.2001.113989 10.1067/mcp.2001.113989 11240971

[2] FDA-NIH Biomarker Working Group (2016) BEST (biomarkers, endpoints, and other tools) resource. Food and Drug Administration (US), Silver Spring (MD) 27010052

[3] Chauvie S, Mazzoni LN, O’Doherty J (2023) A review on the use of imaging biomarkers in oncology clinical trials: quality assurance strategies for technical validation. Tomogr Ann Arbor Mich 9:1876–1902. 10.3390/tomography9050149 10.3390/tomography9050149 PMC10610870 37888741

[4] O’Connor JPB, Aboagye EO, Adams JE et al (2017) Imaging biomarker roadmap for cancer studies. Nat Rev Clin Oncol 14:169–186. 10.1038/nrclinonc.2016.162 27725679 10.1038/nrclinonc.2016.162 PMC5378302

[5] Radiological Society of North America (RSNA) (2025) RSNA—Radiological Society of North America. RSNA

[6] European Society of Radiology (ESR) (2025) MyESR—European Society of Radiology. ESR

[7] Hillman BJ, Gatsonis C (2008) The American College Of Radiology Imaging Network-clinical trials of diagnostic imaging and image-guided treatment. Semin Oncol 35:460–469. 10.1053/j.seminoncol.2008.07.010 18929145 10.1053/j.seminoncol.2008.07.010 PMC3461318

[8] American College of Radiology (ACR) (2025) ACR—American College of Radiology. ACR

[9] Wilkinson MD, Dumontier M, Aalbersberg IjJ et al (2016) The FAIR guiding principles for scientific data management and stewardship. Sci Data 3:160018. 10.1038/sdata.2016.18 26978244 10.1038/sdata.2016.18 PMC4792175

[10] Boss MA, Malyarenko D, Partridge S et al (2024) The QIBA profile for diffusion-weighted MRI: apparent diffusion coefficient as a quantitative imaging biomarker. Radiology 313:e233055. 10.1148/radiol.233055 39377680 10.1148/radiol.233055 PMC11537247

[11] U.S. Food and Drug Administration (2025) Biomarker qualification program. FDA

[12] European Medicines Agency (2025) Biomarker qualification

[13] van Griethuysen JJM, Fedorov A, Parmar C et al (2017) Computational radiomics system to decode the radiographic phenotype. Cancer Res 77:e104–e107. 10.1158/0008-5472.CAN-17-0339 29092951 10.1158/0008-5472.CAN-17-0339 PMC5672828

[14] Generalitat Valenciana (GVA) (2025) ACIM—Área Clínica de Imagen Médica. Available at: https://www.acim.lafe.san.gva.es/acim/?page_id=675&lang=es (Accessed: 15 October 2025).

[15] Instituto de Salud Carlos III (2025) RICORS-REI—Red de Infraestructuras Científicas y Técnicas, proyectos REI. Available at: https://ricors-rei.net/ (Accessed: 15 October 2025).

[16] Martí-Bonmatí L (2021) Evidence levels in radiology: the insights into imaging approach. Insights Imaging 12:45. 10.1186/s13244-021-00995-7 33826000 10.1186/s13244-021-00995-7 PMC8026783

[17] Oto A, Zhu F, Kulkarni K et al (2009) Evaluation of diffusion-weighted MR imaging for detection of bowel inflammation in patients with Crohn’s disease. Acad Radiol 16:597–603. 10.1016/j.acra.2008.11.009 19282206 10.1016/j.acra.2008.11.009 PMC2721917

[18] Knappe L, Bregenzer C, Gözlügöl N et al (2023) New thresholds in semi-quantitative [18F]FDG PET/CT are needed to assess large vessel vasculitis with long-axial field-of-view scanners. Eur J Nucl Med Mol Imaging 50:3890–3896. 10.1007/s00259-023-06423-w 37676501 10.1007/s00259-023-06423-w PMC10611821

[19] Sullivan DC, Obuchowski NA, Kessler LG et al (2015) Metrology standards for quantitative imaging biomarkers. Radiology 277:813–825 26267831 10.1148/radiol.2015142202 PMC4666097

[20] Martí-Bonmatí L, Alberich-Bayarri A (2016) Imaging biomarkers: development and clinical integration. Springer

[21] de Rooij M, Allen C, Twilt JJ et al (2024) PI-QUAL version 2: an update of a standardised scoring system for the assessment of image quality of prostate MRI. Eur Radiol 34:7068–7079. 10.1007/s00330-024-10795-4 38787428 10.1007/s00330-024-10795-4 PMC11519155

[22] Marziali S, Corradini L, Depretto C et al (2026) Assessing breast MRI image quality: the bMRI-QUAL scoring system. Eur Radiol. 10.1007/s00330-026-12439-1 10.1007/s00330-026-12439-1 41824041

[23] Woo S, Luk L, Muglia VF et al (2026) VI-RADS quality score: development and proposal of a scoring system by the American College of Radiology VI-RADS Steering Committee. Eur Radiol 36:1093–1104. 10.1007/s00330-025-11966-7 40830701 10.1007/s00330-025-11966-7

[24] Katz R (2004) Biomarkers and surrogate markers: an FDA perspective. NeuroRx 1:189–195. 10.1602/neurorx.1.2.189 15717019 10.1602/neurorx.1.2.189 PMC534924

[25] Thakur RS, Chatterjee S, Yadav RN, Gupta L (2023) Chapter 5—medical image denoising using convolutional neural networks. In: Rajput SS, Khan NU, Singh AK, Arya KV (eds) Digital image enhancement and reconstruction. Academic Press, pp 115–138

[26] Yu H, Hai Y, Lu J (2025) Exploratory high *b* value diffusion-weighted MR for quantitative differentiation of ileocecal inflammatory conditions and tumors. Insights Imaging 16:41. 10.1186/s13244-025-01916-8 39962029 10.1186/s13244-025-01916-8 PMC11833009

[27] Thormann M, Melekh B, Bär C et al (2023) Apparent diffusion coefficient for assessing Crohn’s disease activity: a meta-analysis. Eur Radiol 33:1677–1686. 10.1007/s00330-022-09149-9 36169687 10.1007/s00330-022-09149-9 PMC9935736

[28] Grayson PC, Alehashemi S, Bagheri AA et al (2018) 18) F-fluorodeoxyglucose-positron emission tomography as an imaging biomarker in a prospective, longitudinal cohort of patients with large vessel vasculitis. Arthritis Rheumatol Hoboken NJ 70:439–449. 10.1002/art.40379 10.1002/art.40379 PMC5882488 29145713

[29] Slart RHJA, Slart RHJA, Glaudemans AWJM et al (2018) FDG-PET/CT(A) imaging in large vessel vasculitis and polymyalgia rheumatica: joint procedural recommendation of the EANM, SNMMI, and the PET Interest Group (PIG), and endorsed by the ASNC. Eur J Nucl Med Mol Imaging 45:1250–1269. 10.1007/s00259-018-3973-8 29637252 10.1007/s00259-018-3973-8 PMC5954002

[30] Martini K, Baessler B, Bogowicz M et al (2021) Applicability of radiomics in interstitial lung disease associated with systemic sclerosis: proof of concept. Eur Radiol 31:1987–1998. 10.1007/s00330-020-07293-8 33025174 10.1007/s00330-020-07293-8 PMC7979612

[31] Haga A, Iwasawa T, Misumi T et al (2024) Correlation of CT-based radiomics analysis with pathological cellular infiltration in fibrosing interstitial lung diseases. Jpn J Radiol 42:1157–1167. 10.1007/s11604-024-01607-2 38888852 10.1007/s11604-024-01607-2 PMC11442537

[32] Song Y, Ma J, Chu T et al (2025) Application of vascular index based on superb microvascular imaging technique for assessing disease activity in rheumatoid arthritis patients with signal-positive joints. Clin Exp Rheumatol 43:1554–1560. 10.55563/clinexprheumatol/8x1nhn 40314989 10.55563/clinexprheumatol/8x1nhn

[33] Naredo E, Möller I, Cruz A et al (2008) Power doppler ultrasonographic monitoring of response to anti-tumor necrosis factor therapy in patients with rheumatoid arthritis. Arthritis Rheum 58:2248–2256. 10.1002/art.23682 18668537 10.1002/art.23682

