# Correction: Toward a standardized and interoperable imaging biomarker catalog

**DOI:** 10.1186/s13244-026-02347-9

**Published:** 2026-07-16

**Authors:** Pablo Rodríguez-Belenguer, Paula Doria-Borrell, Ángel Alberich-Bayarri, Raquel Perez-Lopez, Vicky Goh, Konstantin Nikolaou, Aad van der Lugt, Marion Smits, Luis Rodríguez-Rodríguez, Leonor Cerdá-Alberich, Luis Martí-Bonmatí

**Affiliations:** 1 Biomedical Imaging Research Group (GIBI230), Instituto de Investigación Sanitaria La Fe (IIS La Fe), Valencia, Spain; 2 Quibim—Quantitative Imaging Biomarkers in Medicine, Valencia, Spain; 3 Vall d’Hebron Institute of Oncology (VHIO), Barcelona, Spain; 4 Department of Cancer Imaging, School of Biomedical Engineering and Imaging Sciences, King’s College London, London, United Kingdom; 5 Department of Radiology, University of Tuebingen, Tuebingen, Germany; 6 Department of Radiology and Nuclear Medicine, Erasmus MC University Medical Center, Rotterdam, the Netherlands; 7 Brain Tumor Center, Erasmus MC Cancer Institute, Rotterdam, the Netherlands; 8 Musculoskeletal Pathology Group, Rheumatology Department, Hospital Clínico San Carlos, Instituto de Investigación Sanitaria del Hospital Clínico San Carlos (IdISSC), Madrid, Spain

**Correction to: Insights into Imaging**

10.1186/s13244-026-02304-6,published online 09 June 2026

In this article, the author’s name, Konstantin Nikolaou, was incorrectly written as Konstantin Nikolau.

The original article has been corrected.

