# Supplementary material for: Toward a standardized and interoperable imaging biomarker catalog
Source: Insights Imaging. 2026 Jun 9;17:156. doi: 10.1186/s13244-026-02304-6 (PMC13250019; doi:10.1186/s13244-026-02304-6)
Supplement: Supplementary file 1 — Supplementary information [file 13244_2026_2304_MOESM1_ESM.pdf]

Toward a standardized and interoperable Imaging Biomarker Catalog

ELECTRONIC SUPPLEMENTARY MATERIAL

Supplementary Table 1. Excluded descriptors with their definition and reason of exclusion.

**Excluded descriptors**		
**Descriptor**	**Definition**	**Reason of exclusion**
Biomarker category	Classification based on the nature of the biomarker output and its level of numerical interpretation.	This classification can be inferred from the *Units* and *Range(s)* descriptors; including it explicitly would introduce redundancy without adding discriminatory value.
Susceptibility/risk	Ability of a biomarker to indicate the likelihood of developing a disease or condition before clinical manifestation.	It pertains to individuals without clinical manifestation; since the catalogue focuses on characterized disease states, this category falls outside the defined Context of Use (COU). [10.1177/1535370217750088]
Monitoring	Use of a biomarker for longitudinal assessment of disease status or progression over time.	Monitoring reflects a *temporal application* of a biomarker rather than an intrinsic biomarker characteristic and therefore falls outside the scope of catalogue descriptors.
Pharmacodynamics	How a biomarker changes in response to a specific treatment or intervention.	This classification originates primarily from regulatory and drug-development frameworks and is not universally applicable as an imaging biomarker descriptor.
Bias	Systematic deviation of biomarker measurements from a reference or ground truth.	Bias is a performance or validation characteristic, not a defining feature of an imaging biomarker within a descriptive catalogue.
Linearity	Degree to which biomarker values change proportionally with the underlying biological quantity being measured.	Linearity is a measurement property relevant to analytical validation, rather than a core descriptor of the biomarker itself. [10.1177/0962280214537344]
Commutability	Extent to which biomarker measurements are comparable across platforms, protocols, or institutions.	This is a highly specialized analytical concept applicable only to a limited subset of biomarkers, and therefore not suitable as a general catalogue descriptor.[10.1148/radiol.2015142202]
Reproducibility	Degree to which biomarker measurements are consistent across repeated scans or operators.	Biomarkers are multiparametric, with no current consensus on how to measure reproducibility across different platforms [10.1016/j.acra.2022.08.031]
Sensitivity / specificity	Diagnostic performance metrics for classifying disease or condition.	Reflects clinical performance rather than descriptive attributes; captured indirectly by *Clinical relevance* if needed.
Predictive value (PPV / NPV)	Probability that biomarker correctly predicts disease presence or outcome.	The catalogue is a technical inventory, not a clinical validation study [10.1016/j.acra.2022.08.031]

Robustness	Resistance of biomarker measurement to variations in acquisition, processing, or patient factors.	Robustness is a result of thousands of possible combinations of software settings. There is no standard preprocessing pipeline. [10.3390/cancers16152668]
Normalization / standardization method	Steps applied to harmonize biomarker values across studies or platforms.	Procedural/technical implementation, not a core descriptor
Segmentation method	Method used to identify regions of interest for biomarker extraction.	The interaction between the segmentation boundaries and the normalization shifts creates a “moving target” for measurement. [10.1016/j.ejrad.2025.112086]
Post-processing filters / corrections	Algorithms applied to improve image quality or reduce artifacts.	This descriptor cannot be isolated as a standalone descriptor because its impact is entirely dependent on the preceding steps in the pipeline [10.3390/cancers16152668]

Supplementary Table 2. Included descriptors with their reason of inclusion.

**Included descriptors**	
**Variables**	**Reason of inclusion**
**Imaging biomarker name**	Serves as the unique identifier of the biomarker; essential for cataloguing and referencing.
**Surrogation**	Provides clinical context for the biomarker and its relevance to patient-centered endpoints.
**Clinical relevance**	Helps users understand why the biomarker matters and informs interpretation.
**Main target**	Clarifies intended clinical use, which is central to organizing and interpreting biomarkers.
**Organ(s)**	Supports specificity of biomarker application and enables filtering by organ/system.
**Disease / Substrate**	Connects the biomarker to relevant clinical conditions, enhancing usability of the catalogue.
**Image modality**	Necessary to understand technical basis and applicability of the biomarker.
**Acquisition technique**	Provides reproducibility context; critical for interpreting quantitative biomarker values.
**Technical parameters**	Ensures clarity about how measurements were obtained and supports reproducibility
**Extraction**	Defines how the biomarker is derived and supports consistency and reproducibility.
**Association type**	Differentiates biomarkers by derivation method, which affects interpretability and generalizability.
**Dimensionality**	Indicates complexity of the biomarker and supports understanding of data integration.
**Units**	Provides unambiguous quantification and allows comparison across studies.

**Range(s)**	Supports clinical interpretation and decision-making by defining meaningful cut-offs.
**Actionability**	Links biomarker to potential decisions, enhancing translational relevance.
**Endorsed by publications**	Establishes credibility and evidence base, which is essential for a reliable catalogue.
**Endorsed by professional societies**	Provides authoritative validation and clinical acceptance.
**Regulatory qualifications**	Indicates compliance with regulatory frameworks, relevant for translation to clinical use.
**Repository**	Supports reproducibility and transparency of biomarker derivation.
**Version/author**	Ensures traceability and version control of catalogue content.
